# Genome Sequence of *Pseudomonas* sp. Strain P482, a Tomato Rhizosphere Isolate with Broad-Spectrum Antimicrobial Activity

**DOI:** 10.1128/genomeA.00394-14

**Published:** 2014-06-26

**Authors:** Dorota M. Krzyzanowska, Adam Ossowicki, Sylwia Jafra

**Affiliations:** Laboratory of Biological Plant Protection, Intercollegiate Faculty of Biotechnology UG&MUG, University of Gdansk, Gdansk, Poland

## Abstract

The tomato rhizosphere isolate *Pseudomonas* sp. strain P482 is a member of a diverse group of fluorescent pseudomonads. P482 produces a yet unidentified broad-spectrum antimicrobial compound(s), active *inter alia* (i.a.) against *Dickeya* spp. Here, we present a nearly complete genome of P482 obtained by a hybrid assembly of Illumina and PacBio sequencing data.

## GENOME ANNOUNCEMENT

*Pseudomonas* spp. are Gram-negative aerobic gammaproteobacteria. Many plant-associated species can be found within this genus, both those that are pathogenic and beneficial to plants. The plant-associated pseudomonads produce a set of secondary metabolites, extensively reviewed by Gross and Loper (1). Despite the long-lasting interest of researchers in these microorganisms, strains with new properties are continuously isolated due to high genomic diversity (2).

*Pseudomonas* sp. strain P482 was isolated from the rhizosphere of a garden-cultivated tomato (*Lycopersicon esculentum* Mill.) in Gdynia, Poland. The strain shows strong *in vitro* antagonism toward plant-pathogenic bacteria and fungi, namely *Dickeya* spp., *Pectobacterium* spp. (3), and *Rhizoctonia solani*. In order to aid our research on the background of this antagonism, as well as to enable more detailed studies on the taxonomic affiliation of P482, we have sequenced the genome of the strain.

The nearly complete genome of P482 was obtained in a cost-efficient manner by a hybrid assembly of sequencing data from the Illumina HiSeq and PacBio RS platforms. Sequencing and data processing were performed at BaseClear (Leiden, The Netherlands). Illumina FASTQ reads were assembled using the CLC Genomics Workbench version 5.5.1 (CLC bio, Aarhus, Denmark). The resulting 90 contigs of a total length of 5.615 Mbp (248× coverage) were further concentrated into 52 scaffolds with the SSPACE Premium scaffolder version 2.2 (4) and GapFiller version 1.11 (5). The data from the PacBio instrument were processed using the SMRT Analysis software suite. The continuous long-read (CLR) data were filtered by read length (>50 bp), subread length (>50 bp), and read quality (>0.75), according to a quality analysis of the reads with the CLC Genomics Workbench version 6.5. The assembly of data from both platforms, aimed at creating superscaffolds, was performed using a modified version of the SSPACE Premium scaffolder version 2.3 (4). The orientation, order, and distance between the contigs were determined based on the alignment of the PacBio CLR sequences, performed with BLASR (6).

The hybrid assembly yielded a total of 4 scaffolds: two superscaffolds containing most of the strain genome (3,158,615 bp and 2,486,679 bp) and two much shorter sequences (3,128 bp and 301 bp). The 4 scaffolds were automatically annotated using the IGS Annotation Engine (7) (Institute for Genome Sciences, University of Maryland School of Medicine, USA) (http://ae.igs.umaryland.edu/cgi/index.cgi). The Manatee tool was used for viewing the data (http://manatee.sourceforge.net).

The draft genome of *Pseudomonas* sp. P482 comprises 5,648,723 bp, with an average G+C content of 62.38%. The annotation revealed 5,221 open reading frames (ORFs), 2 rRNA gene operons, and 62 tRNA genes. From the identified ORFs, 4,378 were assigned functions (83.9%).

The genomic sequence was used to investigate the phylogenetic position of *Pseudomonas* sp. P482. The results indicate that P482 represents a novel species (A. Ossowicki, unpublished data). The biochemical and *in silico* studies on antimicrobial metabolites produced by *Pseudomonas* sp. P482 are advanced and will be published elsewhere.

### Nucleotide sequence accession numbers.

This whole-genome shotgun project has been deposited at DDBJ/EMBL/GenBank under the accession no. JHTS00000000. The version described in this paper is version JHTS01000000.
